# Detection of *Staphylococcus aureus* Delta-Toxin Production by Whole-Cell MALDI-TOF Mass Spectrometry

**DOI:** 10.1371/journal.pone.0040660

**Published:** 2012-07-06

**Authors:** Julie Gagnaire, Olivier Dauwalder, Sandrine Boisset, David Khau, Anne-Marie Freydière, Florence Ader, Michèle Bes, Gerard Lina, Anne Tristan, Marie-Elisabeth Reverdy, Adrienne Marchand, Thomas Geissmann, Yvonne Benito, Géraldine Durand, Jean-Philippe Charrier, Jerome Etienne, Martin Welker, Alex Van Belkum, François Vandenesch

**Affiliations:** 1 Hospices Civils de Lyon, Centre National de Référence des Staphylocoques, Centre de Biologie et de Pathologie Est, Bron, France; 2 Université de Lyon, Domaine de la Buire, Lyon, France; 3 INSERM U851, Bacterial Pathogenesis and Innate Immunity laboratory, Lyon, France; 4 Hospices Civils de Lyon, Service de Maladies Infectieuses, Lyon, France; 5 Laboratoire de Chimie et Microbiologie de l’Eau - UMR 6008 CNRS, IBMIG - UFR Sciences Fondamentales et Appliquées, Université de Poitiers, Poitiers, France; 6 bioMérieux S.A, Microbiology Research & Development unit, La Balme Les Grottes, France; 7 bioMérieux S.A, Technology Research Department, Technology platform, Marcy L’Etoile France; National Institutes of Health, United States of America

## Abstract

The aim of the present study was to detect the *Staphylococcus aureus* delta-toxin using Whole-Cell (WC) Matrix Assisted Laser Desorption Ionization - Time-of-Flight (MALDI-TOF) mass spectrometry (MS), correlate delta-toxin expression with accessory gene regulator (*agr*) status, and assess the prevalence of *agr* deficiency in clinical isolates with and without resistance to methicillin and glycopeptides. The position of the delta-toxin peak in the mass spectrum was identified using purified delta-toxin and isogenic wild type and mutant strains for *agr-rna*III, which encodes delta-toxin. Correlation between delta-toxin production and *agr* RNAIII expression was assessed by northern blotting. A series of 168 consecutive clinical isolates and 23 unrelated glycopeptide-intermediate *S. aureus* strains (GISA/heterogeneous GISA) were then tested by WC-MALDI-TOF MS. The delta-toxin peak was detected at 3005±5 Thomson, as expected for the naturally formylated delta toxin, or at 3035±5 Thomson for its G10S variant. Multivariate analysis showed that chronicity of *S. aureus* infection and glycopeptide resistance were significantly associated with delta-toxin deficiency (*p* = 0.048; CI 95%: 1.01–10.24; *p* = 0.023; CI 95%: 1.20–12.76, respectively). In conclusion, the *S. aureus* delta-toxin was identified in the WC-MALDI-TOF MS spectrum generated during routine identification procedures. Consequently, *agr* status can potentially predict infectious complications and rationalise application of novel virulence factor-based therapies.

## Introduction

*Staphylococcus aureus* is an opportunistic pathogen occurring in the nasal cavity of ca. 30% of community-dwelling individuals [1]. It is responsible for a large diversity of diseases including suppurative and toxin-associated infections [2]. *S. aureus* produces numerous virulence factors the expression of which is regulated *in vivo* and *in vitro* by the accessory gene regulator (*agr*) system of which AgrA directly regulates the expression of several phenol soluble modulin (PSM) genes and genes involved in metabolism [3]. The multifunctional regulatory RNAIII is the main effector of the *agr* system [4] and it controls the switch between the expression of surface proteins and exotoxins [5]. In addition, RNAIII is also a messenger RNA for the 26 amino acids (AA) delta-toxin peptide, whose production is a surrogate marker of *agr* functionality [6]. Because of its impact on virulence factor expression, the *agr* system and its effector RNAIII were thought to be of major importance in pathogenesis. Indeed, the vast majority of clinical isolates from acute infections have a functional *agr* system and all such strains produce RNAIII both *in vitro* and *in vivo* [7], [8]. However, *agr*-defective mutant strains have been non-exclusively associated with more chronic clinical situations such as those found in cystic fibrosis (CF) patients. *Agr* deficiency also plays a role during persistent bacteraemia in patients with intravascular devices and it is associated with increased mortality among bacteraemic patients [9]–[11]. *Agr* deficiency has been associated with increased biofilm formation [12] and reduced susceptibility to glycopeptides [13]–[16].

Whole cell (WC) Matrix Assisted Laser Desorption Ionization Time-Of-Flight Mass Spectrometry (MALDI-TOF MS) allows for classification and identification of micro-organisms through proteomic fingerprinting and comparison of the spectra with those in a reference database [17]. MALDI-TOF MS is also a straightforward and reproducible tool for rapidly scanning a variety of other proteomes, detecting and distinguishing hundreds of proteins and peptides in seconds [18]. Hence, several studies have attempted to exploit the WC-MALDI-TOF MS spectrum to detect virulence factors and resistance markers [19], but in the case of *S. aureus* virulence factors, the proof of concept remains to be made [19], [20].

The aim of the present study was to show that routine use of WC-MALDI-TOF MS produces reliable and (clinically) useful information with respect to *S. aureus agr* status. To this end, we have identified the spectral peaks corresponding to the delta-toxin in the MALDI-TOF MS spectrum produced during the routine MS-mediated microbiological identification process [21]. We have next assessed the production of delta-toxin in a collection of consecutive clinical isolates and correlated the results obtained with the clinical context.

**Figure 1:**
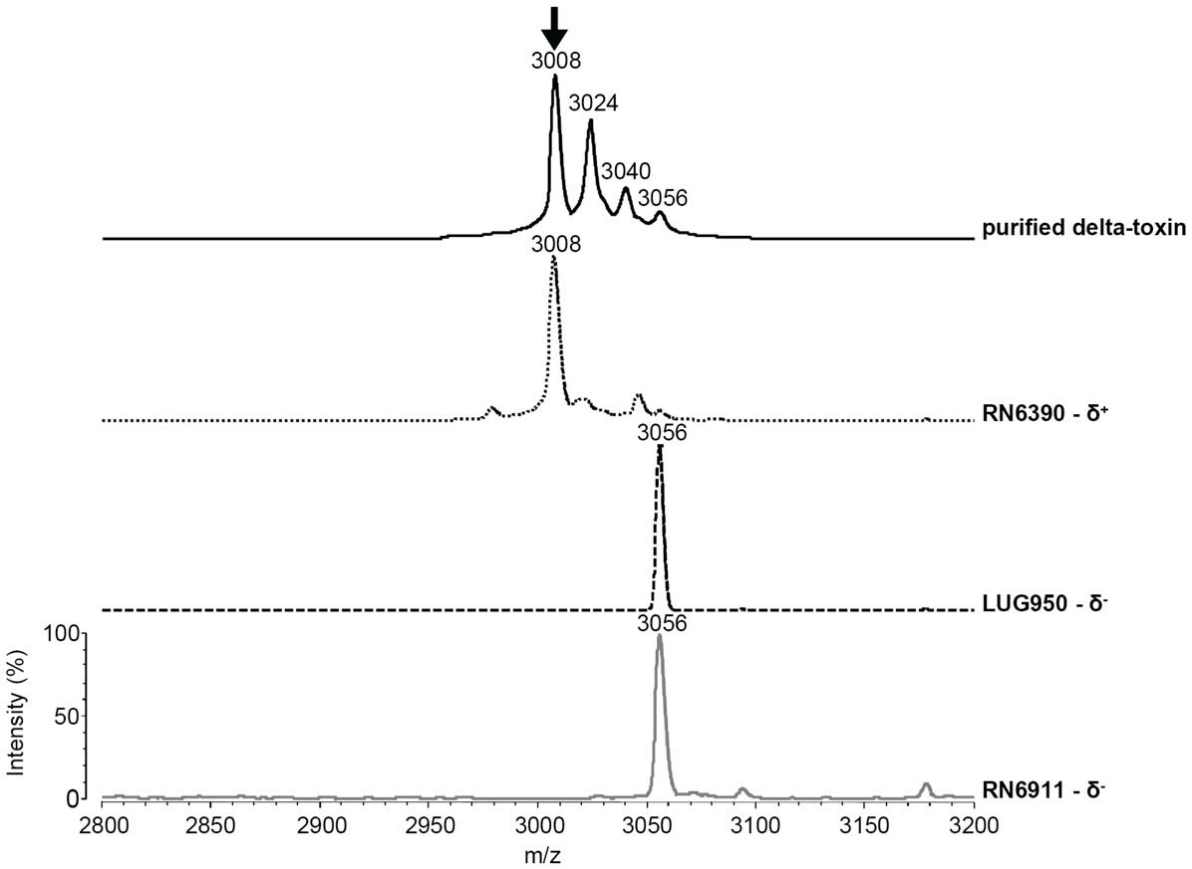
Identification of the delta-toxin peak using purified delta-toxin and isogenic strains. Purified delta-toxin from wild-type *Staphylococcus aureus* (500 ng) or strains mutated in the *accessory gene regulator (agr)* locus were spotted on the target and analysed in the Matrix Assisted Laser Desorption Ionization – Time-of-Flight mass spectrometer with a mass-to-charge ratio (*m*/*z*) range of 2800 to 3200 Thomson (Th). Setting were as follows: mass range 2000–20000 Th; laser power 80 Volts; pulse extraction 8330 Th; number of laser fires per sample 500; noise cut off 10 mVolts with a minimum resolution of 300; Auto quality mode activated. The settings of the detector were according to the linear mode with a positive source of 20000 Th and a negative pulsed extraction at 2100 Th. The arrow in panel A indicates delta-toxin; additional peaks of 3024, 3040 and 3056 Th correspond to contaminants resulting from the purification of the toxin from *S. aureus* culture supernatant. Isogenic strains were: RN6390 (delta positive strain) RN6911 (full *agr knock-out*, delta negative strain) and LUG 950 (*rna*III *knock-out*, delta negative strain).

## Results

### Identification of the Delta-toxin Peak Using Purified Delta-toxin and Isogenic Strain Pairs

Purified delta-toxin (1fg to 500 ng) from *S. aureus* was analysed by MALDI-TOF MS in the range of 2800 to 3200 Thomson (Th). It produced a peak at 3005±1 Th, in the range of the expected weight of the formylated delta-toxin (3005.3 Th for the monoprotonated ion) (Figure 1). This mass is in agreement with previous experimental data [22]. The specific peak was first detected at 1 ng and the peak intensity increased in a dose-dependent manner. Isogenic strains for *agr-rnaIII* (and thus for *hld*) obtained by allelic replacement of the *agr* locus were then spotted on MALDI TOF MS target plates and tested using the “smear method” of deposition. The two *agr*-negative derivatives differed from their parental strain by the lack of the peak at 3005±5 Th (Figure 1).

### Analysis of Robustness and Repeatability of the Method Under Routine Calibration Conditions

The three isogenic strain pairs and two clinical isolates (one delta-toxin positive and one delta-toxin negative) were tested in duplicate after incubation for 18, 24, and 48 hours on trypticase soy agar with horse blood (TSH). The calibration was as used in routine practice (m/z range of 2000 to 20000 Th). In all cases, and at all time points, the 3005±5 Th peak was detected in the delta-toxin-positive strains, and absent in the delta-toxin-negative strains. However, relative intensity of the signal corresponding to the 3005±5 Th tended to slightly decrease at 48 h (see Figure S1). To determine whether the culture media could affect the detection of delta-toxin, several different agar media (Columbia agar with 5% sheep blood (COS), chocolate agar with Polyvitex® mixture (PVX), *S. aureus* chromogenic medium (CHROM ID SA®) and GP agar plate (GP)) were compared for the same 5 strains at 24 h of incubation. These changes in growth conditions did not affect the results (data not shown).

### Analysis of Clinical Strains for the Presence of the Delta-toxin-specific Peak

**Figure 2:**
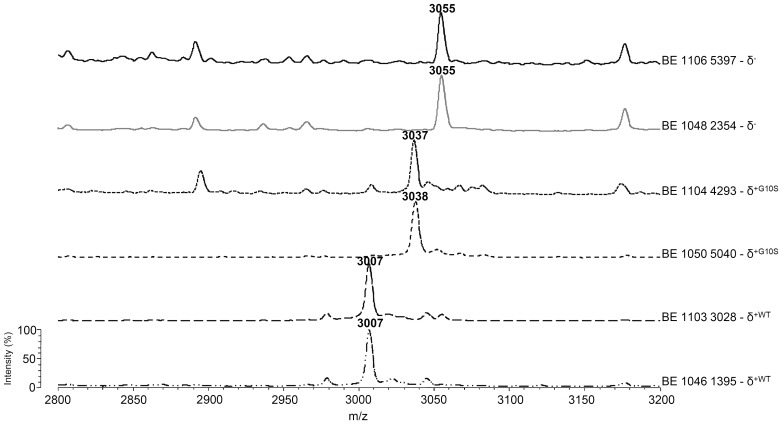
Delta-toxin peak in spectra from clinical strains. Whole-Cell Matrix Assisted Laser Desorption Ionization – Time-of-Flight mass spectrometry analysis of 6 representative clinical strains from the collection of 168. BE1103 3028 and BE1046 1395 are two delta-toxin positive strains showing 3005±5 Thomson (Th) peak; BE1104 4293 and BE1050 5040 are 2 strains expressing a mutated G10S delta-toxin; they exhibited no peak at 3005±5 Th but an additional peak at 3035±5 Th; BE1106 5397 and BE1048 2354 are two delta-toxin negative strains showing no peak at 3005±5 Th or at 3035±5 Th. Settings of the mass spectrometer are the same as in Figure 1.

**Figure 3:**
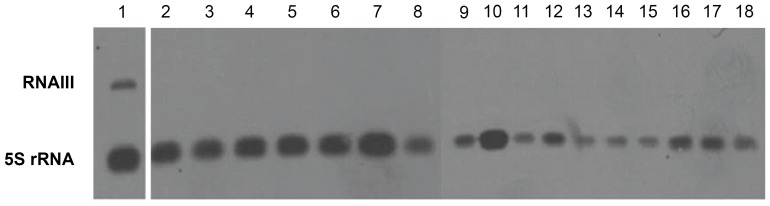
Northern blot analysis of *accessory gene regulator*-RNAIII. Lane 1, positive control isolate (delta-toxin producer); lanes 2 to 18 correspond to the 17 delta-toxin negative clinical isolates. 5S rRNA was used as loading control.

For all clinical isolates collected between November 2010 and March 2011 in our hospitals, the presence of the 3005±5 Th peak was assessed by analyzing the spectra produced during routine identification of these isolates. After removal of duplicates, 168 isolates were retained, of which 139 presented the 3005±5 Th peak, 12 presented a peak at 3035±5 Th, and 17 did not present any peak in this range (Table 1). No synergistic hemolysis was observed for the 17 isolates showing neither 3005±5 Th nor 3035±5 Th peaks. Conversely, a synergistic hemolysis was observed for all 3005±5 Th positive isolates tested and also for the 3035±5 Th positive isolates, suggesting that the 3035±5 Th peak probably corresponded to an allelic variant of delta-toxin (Figure 2). The delta-toxin gene (*hld*) of seven of these isolates was sequenced and showed in all cases a substitution of glycine at position 10 replaced by a serine (G10S), a mutation that changes the expected molecular weight of delta-toxin to 3034.8 Th [23]–[25]. To further confirm the identity of the two specific peaks, a MALDI Time-of-Flight/Time-of-Flight MS (MALDI-TOF/TOF MS) analysis and a search in the UniProtKB/SwissProt database using Mascot® were performed on selected isolates (Table 1). The results confirmed the assignment of the 3005±5 Th to wild type monoprotonated delta-toxin (3005.3 Th) with 86.3 as Mascot score and the 3035±5 Th peak to G10S mutated monoprotonated delta-toxin (3035.8 Th) with 157.4 as Mascot score (Table 1). To ensure that the lack of delta-toxin production in strains lacking both the 3005±5 Th and the 3035±5 Th peaks corresponds to strains defective in *agr* function, Northern blot targeting *agr*-RNAIII was performed on selected isolates. In all cases, these isolates lacked RNAIII expression (Figure 3). Finally, to ensure that the delta-toxin negative isolates did not correspond to a single clone, genotyping of these strains was performed by micro-arrays [26]. It showed that the 17 strains belong to 3 *agr* types and 4 clonal complexes. Of note, the Lyon Clone (ST8, SCC*mec*IV) which is the most prevalent hospital-acquired (HA) methicillin resistant *S. aureus* (MRSA) in France [27] accounted for 6 of the 17 isolates. Genotyping analysis was also performed for 10 of the 12 isolates presenting a peak at 3035±5 Th (two isolates were lost during subculture); it showed that they belong to 4 clonal complexes (CC1, 2 isolates; CC45, 1 isolate; CC59, 4 isolates; and CC121, 3 isolates).

**Table 1:** Determination of delta-toxin status of clinical strains.

	Delta-toxin-specific peak (m/z)		
	Strains with peak at 3005±5 Th^a^(n positive/n analysed)	Strains with peak at 3035±5 Th(n positive/n analysed)	Strains with no peak at 3005±5 and 3035±5 Th (n positive/n analysed)
MALDI-TOF MS^b^	139/168	12/168	17/168
Synergistic hemolysis	139/139	10/10	0/17
*hld* c gene sequence	NT^d^	7^G10S^ e/7	9^WT^ f/9
MALDI/TOF-TOF MS^g^	1^WT^/1	1^G10S^/1	NT
Northern blot *agr*-RNAIII	1/1	NT	0/17

a Th: Thomson.

b MALDI-TOF MS: Matrix Assisted Laser Desorption Ionization Time-Of-Flight mass spectrometry.

c *hld*: gene encoding delta-toxin.

d NT: not tested.

e G10S: mutated delta-toxin with glycine 10 replaced by a serine.

f WT: wild type delta-toxin.

g MALDI/TOF-TOF MS: Matrix Assisted Laser Desorption Ionization Time-Of-Flight/Time-Of-Flight mass spectrometry.

### Links between Clinical Status and Delta-toxin Production

Overall, delta-toxin-deficiency and *agr*-dysfunction in our series was found in 10% (17/168) of the clinical isolates. To determine whether *agr* dysfunction was correlated with chronicity of infection, and/or with the presence of biomaterial infections, the 17 patients with delta-toxin-negative isolates and the 151 patients with delta-positive isolates were categorized according to the clinical presentation of their infection or colonization. The results showed that the lack of delta-toxin production was significantly associated with chronicity of infections (*p* = 0.005); however, there was no significant difference in delta-toxin production between infection and colonisation (*p* = 0.541), and there were no associations with biomaterial infection (*p* = 0.470), or with in-hospital 30 day mortality (*p* = 0.574) (Table 2).

**Table 2:** Microbiological and patient characteristics stratified by *accessory gene regulator* function.

	Number of strains			
Characteristic	Delta-toxin positive(n = 151)	Delta-toxin negative(n = 17)	All strains(n = 168)	*p* value^a^
**Strain characteristics**				
Methicillin resistance	21	6	27	0.035^b^
GISA^c^, hGISA^d^ phenotypes	1	4	5	ND^e^
**Patients characteristics**				
Median age (range)	46 (1mo-94yrs)^f^	19 (1mo-79yrs)	45 (1mo-94yrs)	0.169^g^
Sex ratio (M/F)^h^	1.3 (86/65)	0.88 (8/9)	1.3 (95/73)	0.299^b^
Infection *versus* colonization	118/33	13/4	131/37	0.541^b^
Acute *versus* chronic infection	104/14	7/6	111/20	0.005^b^
Implantable biomedical devices; presence*versus* absence	19/132	3/14	22/146	0.470^b^
Mortality^i^ : non survivors *versus* survivors	13/138	1/16	14/154	0.574^b^

a *p value on univariate analysis.

b Fisher’s exact test.

c GISA: Glycopeptides Intermediate *Staphylococcus aureus*.

d hGISA: heterogeneous GISA.

e ND: not determined.

f mo: month; yrs: years.

g Mann-Whitney test.

h M: male, F:female.

i 30-days mortality.

### Antibiotic Resistance and Delta-toxin Production

Methicillin resistance was significantly associated with the delta-toxin minus status in our series of 168 clinical isolates (univariate analysis, *p* = 0.035) (Table 2). When considering glycopeptide resistance, 5 of the 168 isolates showed a heterogeneous glycopeptide-intermediate *S. aureus* (hGISA) profile by antibiotic susceptibility tests and population analysis. Of those, 4 were delta-toxin deficient and 1 delta-toxin positive (sample size too small for statistical analysis) (Table 2). To investigate further the association between *agr*-dysfunction and glycopeptide resistance, we compared the *agr* status of the 5 hGISA from the above series plus an additional collection of 23 well characterized glycopeptide-intermediate *S. aureus* (GISA) (8 strains) and hGISA (15 strains) from the National Reference Center for Staphylococci, with that of the 126 non-GISA isolates selected from the present series of 168 clinical isolates (after removal of the colonization isolates). The lack of clonality of the GISA/hGISA series was ruled out by genotyping (CC1, 2 isolates; CC5, 10 isolates; CC8, 13 isolates; CC15, 1 isolate; CC59, 1 isolate; CC88, 1 isolate). Delta-toxin production assessed by WC-MALDI TOF MS revealed that 9/28 GISA/hGISA *versus* only 9/126 non-GISA were delta-toxin negative (univariate analysis, *p* = 0.001) (Table 3). Since glycopeptide resistance is classically associated with methicillin resistance and with chronic infections, multivariate analysis was performed on the results obtained for the 126 non-GISA plus the 28 GISA/hGISA. Methicillin resistance, glycopeptide resistance and chronic *versus* acute status of infection were included in the analysis (Table 3). In the multivariate model 1, methicillin resistance was not significantly associated with GISA/hGISA phenotypes and chronic infection and this was confirmed by a non-significant likelihood ratio test. The results showed (Table 3, model 2) that *S.* *aureus* chronic infection and glycopeptide resistance remained independently and significantly associated with delta-toxin deficiency (*p* = 0.048; confidence interval at 95% (CI 95%): 1.01–10.24; *p* = 0.023; CI 95%: 1.20–12.76, respectively).

**Table 3:** Multivariate analysis of *accessory gene regulator* status for 154 infecting isolates.

		*agr* a status		Univariate analysis			Multivariate analysis						
							Model 1			Model 2			Likelihood
		*agr* +	*agr−*	OR^b^	95% CI^c^	*p value*	OR	95% CI	*p value*	OR	95% CI	*p value*	ratio test
GISA/hGISA^d^	–	117	9	1.00^e^	–	–	1.00^e^	–	–	1.00^e^	–	–	*p* = 0.435^f^
	+	19	9	6.16	2.17 – 17.48	0.001	3.06	0.85 – 11.01	0.087	3.83	1.20 – 12.76	0.023	
Methicillinsus-ceptibility	MSSA^g^	113	9	1.00^e^	–	–	1.00^e^	–	–				
	MRSA^h^	23	9	4.44	1.60 – 12.32	0.004	1.67	0.47 – 6.01	0.431				
Infection	Acute	115	9	1.00^e^	–	–	1.00^e^	–	–	1.00^e^	–	–	
	Chronic	21	9	5.48	1.95 –15.41	0.001	2.86	0.87 – 9.40	0.083	3.21	1.01 – 10.24	0.048	

a *agr*: accessory gene regulator;

b OR: odd ratio;

c CI: confidence interval;

d GISA: glycopeptide intermediate *Staphylococcus aureus* strains - hGISA: heterogeneous GISA;

e Reference;

f χ^2^ (1 degree of freedom)  =  -2 log likelihood (model 2 - model 1)  = 0.608; *p* = 0.413;

g MSSA: methicillin-sensitive *Staphylococcus aureus*;

h MRSA: methicillin-resistant *Staphylococcus aureus.*

### Agr Dysfunction is Selected During the Course of Infection

For several of the delta-toxin negative infected patients we had consecutive isolates of *S. aureus* available. We wondered whether the delta-toxin negative isolate derived from a previous delta positive isolate. Genotyping of the delta-toxin isolates previously cultured from the same patients in the preceding weeks or months was performed by microarray probing. For seven cases, a previously cultured delta-toxin positive isolate could be found and their overall genotypes were indistinguishable to that of the delta-toxin negative isolate (Table S1). In two cases, glycopeptide consumption by the patients could be documented. Taken together, this suggests that at least in these 7 cases, a within-host shift of *agr* function has apparently occurred *in vivo*.

## Discussion

The MALDI-TOF MS technology is now recognized as the most efficient method for bacterial identification in routine laboratory practice [21]. Beyond the goal of identification, the use of the proteomic spectra to address clinically relevant questions involving the detection of resistance markers or virulence factors, or performing inter-strain comparison, is a very exciting challenge and a field of growing interest [19], [20], [28]. Although it was previously shown that delta-toxin expression can be measured by liquid chromatography-mass spectrometry (LC-MS) [29], the present study demonstrates that the same delta-toxin could be easily recognized by WC-MALDI-TOF mass spectrometry as used in routine practice. We confirmed that the lack of expression of delta-toxin is correlated with dysfunction of *agr*, the major *S. aureus* virulence regulon, and that this dysfunction correlates with *S. aureus* chronic infection during CF or as a consequence of diabetic foot; in several cases we could demonstrate that a within-host shift of *agr* function may have occurred *in vivo*. In addition, we confirmed that glycopeptide resistance or hetero-resistance is associated with *agr* dysfunction. This provides a first alert towards a probability of resistance several hours to even more than a day before completion of the classical antibiogram.

We have previously shown that the use of isogenic strains is probably one of the best approaches to ensure that a given peak in the MALDI-TOF MS spectrum may or may not correspond to an expected protein. Here, purified delta-toxin as well as the use of isogenic *knock-out* mutants were essential in the confirmation of the delta-toxin peak in WC-MALDI-TOF MS mass spectra (Figure 1). The use of a large number of routine clinical isolates confirmed the feasibility of the detection and its robustness with respect to culture conditions and incubation time. However, a number of routine clinical isolates appeared to produce a single amino acid-mutant form of delta-toxin (G10S), a mutation initially reported with the fourth *agr* group [25] and subsequently also found in other backgrounds such as MW2 [23] and MSSA476 strains [24]. The functionality of the delta-toxin G10S from MW2 strain has been assessed [30]; however the prevalence of this mutation (7% in the present study) was not known before. Overall, the presence of the 3005±5 Th or the 3035 Th peaks reflected the presence of delta-toxin, and the lack of both correlated with lack of delta-toxin (Table 1). Despite the lack of specificity of the synergistic hemolysis assay [31], all delta-toxin-positive strains tested produced a positive synergy (Table 1).

As far as we know, the current results demonstrate for the first time that identification of a specific toxin within a MALDI-TOF MS spectrum using whole cell preparations is clearly possible. Indeed, previous attempts to correlate the presence of a 4448 Th peak to the expression of the Panton Valentine leukocidin showed that the 4448 Th peak actually was a marker of a strain background (the ST80 community-acquired (CA)-MRSA clone) and not the toxin itself [19], [20]. Delta-toxin production is considered to be a surrogate marker of the functionality of *agr*, nutrient transport, amino acid metabolism and other processes in *S. aureus* [7]. It is generally proposed that full virulence in acute infection requires a functional *agr* system whilst *agr* dysfunction is associated with chronic infections, the formation of small colony variants, and biofilm associated infections with their increased resistance to antibiotics [7], [8], [22], [32].

In addition, *agr* dysfunction has been associated with glycopeptide resistance of GISA or hGISA type [14]–[16], [33]. With this in mind, we first verified by northern blotting for RNAIII (the *agr* effector), that there was a good correlation between delta-toxin detection and RNAIII expression. In fact, there was no doubt that if delta-toxin was present it would be accompanied by RNAIII expression; conversely, there was a theoretical possibility that some delta-toxin negative isolates would be caused by frame shift(s) in the *hld* coding sequence or by translational defects [34]. Actually, all delta-toxin negative isolates were found to be non-producers of RNAIII (Table 1), thus confirming that delta-toxin is a marker of *agr* function. This being established, we tested a series of clinical isolates for the presence of delta-toxin (and thus *agr* function) and found that in a non-selected consecutive series of 168 isolates collected in our university hospital during 6 months, there were 10% of delta-toxin negative isolates, a rate below that published by Traber *et al.* (22.6%) on a series of 146 non-selected infections isolates [8] or the rate of 22.2% of *agr* dysfunction among 814 bacteremia isolates [11]. Strikingly, the rate of delta-toxin-negative isolates was much higher in the series of 39 bacteraemia isolates studied by Fowler *et al.*, with a rate of 38.9% in resolving bacteraemia and 71.4% in chronic bacteraemia [10]. Of note, in our series of 168 patients we had very few bacteraemia isolates, and *agr* dysfunction was observed mainly in chronic lung infections in cystic fibrosis patients and chronic infections in diabetic patients (Table S2). Hence, chronicity appears to be the major factor associated with *agr* dysfunction as assessed by the multivariate analysis which included methicillin resistance, glycopeptide resistance and chronic *versus* acute status of infections (*p* = 0.048; CI 95%: 1.01–10.24) (Table 3). Chronicity as a factor associated with *agr* dysfunction could be documented *in vivo* for 7 patients, by showing that their delta-toxin negative isolate was selected from a previous delta positive population (Table S1). This is in agreement with other reports showing that *agr*-negativity is commonly selected during the course of infections [8]. Multivariate analysis also revealed that glycopeptide resistance was significantly associated with *agr* dysfunction (*p* = 0.02; CI 95%: 1.20–12.76) (Table 3), in accordance with previous studies [14]–[16]. Interestingly, methicillin resistance, which was associated with *agr* dysfunction in the univariate analysis (Table 2), appeared to be a confounding factor in the logistic regression model (Table 3). This reflects the fact that methicillin resistance is associated in Europe with HA infections, which are more frequently chronic than CA-acute infections.

The first limitation of the current study is that all our isolates were tested for delta-toxin production after 24 hours of incubation, a time at which strains with delayed RNAIII production [34] can appear delta toxin positive. Further study using an adapted protocol to perform a WC MALDI TOF analysis at 6 hrs of incubation may increase the proportion of isolates that should be considered as phenotypically *agr*-deficient. The second limitation of the current study is that the clinical data were not collected through a standardized case record form. Thus, the link observed between *agr* deficiency and chronicity of infection -there is not a universal definition of chronicity- should be considered with caution and deserves further study. In addition, the present study was based on a non-selected population in which some important pathologies such as infective endocarditis or chronic bacteraemia were not sufficiently represented to allow statistical analysis and comparison with other studies. The implementation of MALDI-TOF MS as a tool for bacterial identification in a growing number of clinical laboratories will encourage future prospective and possibly multicenter studies centered on those diseases.

In conclusion, we have identified the MS peaks corresponding to the delta-toxin in the MALDI spectrum produced during routine identification process and confirm that routine use of WC MALDI-TOF MS produces reliable and useful information with respect to *agr* status.

## Materials and Methods

### Purification of Delta-toxin

The delta-toxin peptide from *S. aureus* 2850 strain was purified by high-performance liquid chromatography (HPLC) using a two-step purification procedure as described previously [35].

### Bacterial Isolates

#### Isogenic strains

Three isogenic strain pairs were tested to validate the specificity of delta-toxin detection: RN6390 (delta-toxin positive) derives from the NCTC8325-4 strain [36]; RN6911 is the *agr knock-out* (delta-toxin negative) derivative of RN6390 [37]; and LUG950 is a deletion/replacement Δ*rnaIII* (nts1579 to 1015 in the *agr* sequence)/aphA-3 mutant of RN6390 constructed by using pMAD [38].

#### Clinical isolates

A series of 168 *S. aureus* isolates cultured from patient samples in three university hospitals in Lyon totalizing 2200 beds were prospectively collected between November 2010 and March 2011. All isolates were initially identified by conventional biochemical methods as defined by the manufacturer (Phoenix®100 identification system, Becton Dickinson) and WC-MALDI-TOF MS (see below). Clinical data were retrieved for all isolates by using the hospital information system (Cristal Net®, CHU Grenoble). Each case was reviewed by two senior clinical microbiologists (FV, JE) and a senior infectious disease practitioner (FA). *S. aureus* chronic infection was defined as follows: (*i*) persistent bacteraemia as ≥7 days of positive blood cultures despite receiving appropriate antibiotic treatment [39]; (*ii*) chronic surgical-site infection defined as persisting after completion of the antimicrobial therapy duration recommended for the corresponding site of infection, e.g. intra-abdominal infection after a 7 day therapy [40]; (*iii*) chronic device-related infections, e.g. intravascular catheter-related infections as defined in updated management guidelines in case of long-term catheter [41]; (*iv*) adult CF patients with *S. aureus* long-term persistence in lung airways [42]; (v) diabetic foot infection with proven bone infectious focus [43]. The research was conducted in accordance with the ethical chart of the Hôpitaux de Lyon and in accordance with French Government regulation. According to those guidelines our epidemiological study did not require ethics approval and written consent as long as clinical data were analysed anonymously.

#### Glycopeptide intermediate *S. aureus* (GISA) strains

Twenty-eight unrelated GISA strains collected by the French National Reference Center for Staphylococci, throughout the whole French territory between 2008 and 2011 were included. The strains were initially referred for precise assessment of antibiotic resistance. Glycopeptide resistance was assessed by using the modified population analysis protocol described below.

### Bacterial Growth Conditions

*S. aureus* strains were cultivated on trypticase soy agar with horse blood (TSH, bioMérieux) during 24hours at 35±2°C under aerobic conditions. Alternatively, Columbia agar with 5% sheep blood (COS), chocolate agar with Polyvitex® mixture (PVX), *S. aureus* chromogenic medium (CHROM ID SA®) (all from bioMérieux) and GP agar plate (Difco®, Becton Dickinson) were used. Reproducibility and intermediate precision tests were performed.

### MALDI-TOF MS Analyses

#### Mass spectrometer

Analyses were performed on an Axima Assurance® mass spectrometer (Shimadzu Biotech) using conventional settings determined for bacterial identification and piloted by LaunchPad® software (version 2.8.4.20081127, Shimadzu Biotech). Bacterial identification was achieved via database searching (SARAMIS®, bioMérieux) using *Escherichia coli* ATCC 8739 for calibration. Additional calibration was performed in the 2800 - 3200 Th range by using PEG3000 dissolved in matrix solution (Sigma).

#### Analysis

Purified delta-toxin diluted in acetonitril 50%, formic acid 0.1%, purified water 49.9%, was deposited on disposable target plates (Fleximass DS®, bioMérieux) followed by 15 minutes of drying. Then one microliter of mass spectrometry matrix (sinapinic acid, bioMérieux) was added followed by 15 minutes drying.

Bacterial strains were processed by following the protocol used to identify bacteria to the species level by WC-MALDI-TOF MS in routine practice using α-cyano-4-hydroxycinnamic acid (CHCA) matrix [44]. Mass spectral profiles were obtained with the help of LaunchPad® (Shimadzu Biotech) or SirWeb MaldiTof® software (I2A).

### MALDI Time-of-Flight/Time-of-Flight (MALDI-TOF/TOF) MS

One colony of cells from a 24 hrs culture on TSH agar as described above was deposited on a stainless steel target (Bruker Daltonics) using a dried-droplet preparation and CHCA as matrix. Mass spectrometric profiles were recorded in positive reflectron mode using an Ultraflex II MALDI-TOF/TOF mass spectrometer (Bruker Daltonics). Peaks of interest were manually selected and subjected to further analysis using the LIFT® technology in TOF/TOF mode to confirm or disprove protein identifications. Resulting peptide fragmentation mass profiles were analysed using Mascot® software (Matrix Science) and the UniProtKB/SwissProt database (release 2011-07) for peptide identification [45].

### Northern Blot Analysis of *agr*-RNAIII

RNA was extracted from post-exponential (6 hours) agitated Brain Heart Infusion broth (Becton Dickinson)-grown cultures using the RNeasy Plus® Mini kit (Qiagen) with an additional lysostaphin step [46]. Electrophoresis of total RNA was carried out on a 1% agarose gel containing 2.2M formaldehyde, followed by vacuum transfer to a nylon membrane. *agr*-RNAIII and 5S ribosomal RNA were detected by hybridizations with specific digoxigenin-labelled RNA probes and luminescent detection was carried out as described previously [47].

### Assessment of Delta-toxin Expression by Synergistic Hemolysis

Delta-toxin production was determined in an agar plate assay testing synergy with a beta-toxin-producing reference strain (*Staphylococcus intermedius* N890223) after 24 hours and 48 hours of incubation [15].

### Determination of GISA Susceptibility Profiles

Minimal Inhibitory Concentrations (MICs) for vancomycin and teicoplanin were determined by standard Etest® (bioMérieux) using a 0.5 McFarland inoculum on Mueller Hinton agar plates (bioMérieux) incubated at 37°C for 24 hours. Screening for GISA/VISA were determined by Etest® (bioMérieux) using a 2 McFarland inoculum on Brain Heart Infusion agar plates (Difco) incubated at 37°C for 48 hours [48]. Population analysis was conducted according to the method of Hiramatsu *et al* adapted by Wootton *et al* [49]–[50]. Isolates tested were suspended in brain–heart infusion broth to a 2 McFarland standard of which a 200 µL inoculum was plated on BHI agar plates containing 4, 6 and 8 mg/L vancomycin. Plates were incubated at 37 C for 24 and 48 hrs and colonies were counted. GISA *versus* hGISA were categorized according to the definition of Hiramatsu *et al* [49]. Strain Mu3 and Mu50 were used as controls.

### Bacterial Genotyping by DNA-microarray

DNA microarrays (Identibac®, Alere Technologies) were used according to the protocol described by Monecke *et al.* [26]. This DNA microarray detects a total of 332 different target sequences corresponding to 185 distinct genes and their allelic variants. Analysis of the recorded picture was performed using the ArrayMate® reader and software (Alere Technologies). Data interpretations were based on the algorithm previously described [51].

### Statistical Analysis

Fisher’s exact test was used to compare categorical variables (glycopeptide or methicillin resistances, sex, colonisation *versus* infection, acute *versus* chronic conditions, implantable biomedical devices *versus* absence and non-survivor *versus* survivor cases) with *S. aureus* delta-toxin production. The Mann-Whitney *U*-test was used to compare age distribution according to *S. aureus* delta-toxin production.

Univariate and multivariate logistic regression was used to determine whether chronic status, GISA and methicillin resistance were independently associated with delta-toxin production. A likelihood ratio test was used to select the most parsimonious model explaining *S. aureus* delta-toxin production. All analyses were performed using SPSS® software (SPSS version 20 for Mac).

## Supporting Information

Figure S1 **Analysis of robustness and repeatability of the method under routine calibration conditions.** Matrix Assisted Laser Desorption Ionization/Time-Of-Flight mass spectrometry analysis of 3 isogenic strains and 2 representative clinical isolates from the collection of 168. RN6390 (delta-toxin positive parental strain) showing 3005±5 Thomson (Th) peak, RN6911 (full *agr knock-out*, delta-toxin negative isogenic derivative) and LUG950 (*rna*III *knock-out*, delta-toxin negative isogenic derivative) showing no peak at 3005±5 Th or at 3035±5 Th; BE1046 1395 (delta-toxin positive clinical isolate) showing 3005±5 Th peak and BE1048 2354 (delta-toxin negative clinical isolate) showing no peak at 3005±5 Th or at 3035±5 Th. Strains were tested in duplicate after incubation for 18, 24, and 48 hours on trypticase soy agar plates with horse blood. The calibration was that used in routine practice (m/z range of 2000 to 20000 Th).
(TIFF)

Table S1 **Genotyping of 7 couples of** ***Staphylococcus aureus*** **isolates using microarrays.**
(PDF)

Table S2 **Clinical characteristics of patients with chronic infections.**
(PDF)

## References

[1] Bode LG, Wertheim HF, Kluytmans JA, Bogaers-Hofman D, Vandenbroucke-Grauls CM (2011). Sustained low prevalence of meticillin-resistant *Staphylococcus aureus* upon admission to hospital in The Netherlands.. J Hosp Infect.

[2] Li M, Cheung GY, Hu J, Wang D, Joo HS (2010). Comparative analysis of virulence and toxin expression of global community-associated methicillin-resistant *Staphylococcus aureus* strains.. J Infect Dis.

[3] Queck SY, Jameson-Lee M, Villaruz AE, Bach TH, Khan BA (2008). RNAIII-independent target gene control by the *agr* quorum-sensing system: insight into the evolution of virulence regulation in *Staphylococcus aureus*.. Mol Cell.

[4] Novick RP, Geisinger E (2008). Quorum sensing in staphylococci.. Annu Rev Genet.

[5] Felden B, Vandenesch F, Bouloc P, Romby P (2011). The *Staphylococcus aureus* RNome and its commitment to virulence.. PLoS Pathog.

[6] Janzon L, Lofdahl S, Arvidson S (1989). Identification and nucleotide sequence of the delta-lysin gene, *hld*, adjacent to the accessory gene regulator (*agr*) of *Staphylococcus aureus*.. Mol Gen Genet.

[7] Thoendel M, Kavanaugh JS, Flack CE, Horswill AR (2011). Peptide signaling in the staphylococci.. Chem Rev.

[8] Traber KE, Lee E, Benson S, Corrigan R, Cantera M (2008). *agr* function in clinical *Staphylococcus aureus* isolates.. Microbiology.

[9] Goerke C, Campana S, Bayer MG, Doring G, Botzenhart K (2000). Direct quantitative transcript analysis of the *agr* regulon of *Staphylococcus aureus* during human infection in comparison to the expression profile *in vitro*.. Infect Immun.

[10] Fowler VGJ, Sakoulas G, McIntyre LM, Meka VG, Arbeit RD (2004). Persistent bacteremia due to methicillin-resistant *Staphylococcus aureu*s infection is associated with *agr* dysfunction and low-level in vitro resistance to thrombin-induced platelet microbicidal protein.. PLoS Pathog J Infect Dis J Infect Dis.

[11] Schweizer ML, Furuno JP, Sakoulas G, Johnson JK, Harris AD (2011). Increased mortality with *accessory gene regulator* (*agr*) dysfunction in *Staphylococcus aureus* among bacteremic patients.. Antimicrob Agents Chemother.

[12] Vuong C, Saenz HL, Gotz F, Otto M (2000). Impact of the *agr* quorum-sensing system on adherence to polystyrene in *Staphylococcus aureus*.. J Infect Dis.

[13] Harigaya Y, Ngo D, Lesse AJ, Huang V, Tsuji BT (2011). Characterization of heterogeneous vancomycin-intermediate resistance, MIC and *accessory gene regulator* (*agr*) dysfunction among clinical bloodstream isolates of *Staphyloccocus aureus*.. BMC Infect Dis.

[14] Cafiso V, Bertuccio T, Spina D, Purrello S, Blandino G (2012). A novel delta-hemolysis screening method to detect hVISA and VISA.. J Clin Microbiol.

[15] Sakoulas G, Eliopoulos GM, Moellering RC, Wennersten C, Venkataraman L (2002). *Accessory Gene Regulator* (*agr*) Locus in Geographically Diverse *Staphylococcus aureus* Isolates with Reduced Susceptibility to Vancomycin.. Antimicrobial Agents and Chemotherapy.

[16] Tsuji BT, MacLean RD, Dresser LD, McGavin MJ, Simor AE (2011). Impact of *accessory gene regulator* (*agr*) dysfunction on vancomycin pharmacodynamics among Canadian community and health-care associated methicillin-resistant *Staphylococcus aureus*.. Ann Clin Microbiol Antimicrob.

[17] Bergeron M, Dauwalder O, Gouy M, Freydiere AM, Bes M (2011). Species identification of staphylococci by amplification and sequencing of the *tuf* gene compared to the *gap* gene and by matrix-assisted laser desorption ionization time-of-flight mass spectrometry.. Eur J Clin Microbiol Infect Dis.

[18] Fagerquist CK, Garbus BR, Williams KE, Bates AH, Boyle S (2009). Web-based software for rapid top-down proteomic identification of protein biomarkers, with implications for bacterial identification.. Appl Environ Microbiol.

[19] Bittar F, Ouchenane Z, Smati F, Raoult D, Rolain JM (2009). MALDI-TOF-MS for rapid detection of staphylococcal Panton-Valentine leukocidin.. Int J Antimicrob Agents.

[20] Dauwalder O, Carbonnelle E, Benito Y, Lina G, Nassif X (2010). Detection of Panton-Valentine toxin in *Staphylococcus aureus* by mass spectrometry directly from colony: time has not yet come.. Int J Antimicrob Agents.

[21] Seng P, Drancourt M, Gouriet F, La Scola B, Fournier PE (2009). Ongoing revolution in bacteriology: routine identification of bacteria by matrix-assisted laser desorption ionization time-of-flight mass spectrometry.. Clin Infect Dis.

[22] Somerville GA, Cockayne A, Durr M, Peschel A, Otto M (2003). Synthesis and Deformylation of *Staphylococcus aureus* -Toxin Are Linked to Tricarboxylic Acid Cycle Activity.. Journal of Bacteriology.

[23] Voyich JM, Vuong C, DeWald M, Nygaard TK, Kocianova S (2009). The *Sae*R/S gene regulatory system is essential for innate immune evasion by *Staphylococcus aureus*.. J Infect Dis.

[24] Holden MT, Feil EJ, Lindsay JA, Peacock SJ, Day NP (2004). Complete genomes of two clinical *Staphylococcus aureus* strains: evidence for the rapid evolution of virulence and drug resistance.. Proc Natl Acad Sci U S A.

[25] Jarraud S, Lyon GJ, Figueiredo AM, Gerard L, Vandenesch F (2000). Exfoliatin-producing strains define a fourth *agr* specificity group in *Staphylococcus aureus*.. J Bacteriol.

[26] Monecke S, Jatzwauk L, Weber S, Slickers P, Ehricht R (2008). DNA microarray-based genotyping of methicillin-resistant *Staphylococcus aureus* strains from Eastern Saxony.. Clin Microbiol Infect.

[27] Dauwalder O, Lina G, Durand G, Bes M, Meugnier H (2008). Epidemiology of invasive methicillin-resistant *Staphylococcus aureus* clones collected in France in 2006 and 2007.. J Clin Microbiol.

[28] van Belkum, A., Welker M, Erhard M, Chatellier S (2012). Biomedical mass spectrometry in today’s and tomorrow’s clinical microbiology laboratory.. J Clin Microbiol.

[29] Otto M, Gotz F (2000). Analysis of quorum sensing activity in staphylococci by RP-HPLC of staphylococcal delta-toxin.. Biotechniques 28: 1088, 1090, 1092, 1096.

[30] Wang R, Braughton KR, Kretschmer D, Bach TH, Queck SY (2007). Identification of novel cytolytic peptides as key virulence determinants for community-associated MRSA.. Nat Med.

[31] Cheung GY, Duong AC, Otto M (2011). Direct and synergistic hemolysis caused by Staphylococcus phenol-soluble modulins: implications for diagnosis and pathogenesis.. Microbes Infect.

[32] Verdon J, Girardin N, Lacombe C, Berjeaud JM, Hechard Y (2009). delta-hemolysin, an update on a membrane-interacting peptide.. Peptides.

[33] Sakoulas G, Eliopoulos GM, Fowler VGJ, Moellering RCJ, Novick RP (2005). Reduced susceptibility of *Staphylococcus aureus* to vancomycin and platelet microbicidal protein correlates with defective autolysis and loss of *accessory gene regulator* (*agr*) function.. Antimicrob Agents Chemother.

[34] Traber K, Novick R (2006). A slipped-mispairing mutation in AgrA of laboratory strains and clinical isolates results in delayed activation of agr and failure to translate delta- and alpha-haemolysins.. Mol Microbiol.

[35] Marchand A, Verdon J, Lacombe C, Crapart S, Hechard Y (2011). Anti-Legionella activity of staphylococcal hemolytic peptides.. Peptides.

[36] Kreiswirth BN, Lofdahl S, Betley MJ, O’Reilly M, Schlievert PM (1983). The toxic shock syndrome exotoxin structural gene is not detectably transmitted by a prophage.. Nature.

[37] Peng HL, Novick RP, Kreiswirth B, Kornblum J, Schlievert P (1988). Cloning, characterization, and sequencing of an *accessory gene regulator* (*agr*) in *Staphylococcus aureus*.. J Bacteriol.

[38] Arnaud M, Chastanet A, Debarbouille M (2004). New vector for efficient allelic replacement in naturally nontransformable, low-GC-content, gram-positive bacteria.. Appl Environ Microbiol.

[39] Levine DP, Fromm BS, Reddy BR (1991). Slow response to vancomycin or vancomycin plus rifampin in methicillin-resistant *Staphylococcus aureus* endocarditis.. Ann Intern Med.

[40] Solomkin JS, Mazuski JE, Bradley JS, Rodvold KA, Goldstein EJ (2010). Diagnosis and management of complicated intra-abdominal infection in adults and children: guidelines by the Surgical Infection Society and the Infectious Diseases Society of America.. Clin Infect Dis.

[41] O’Grady NP, Alexander M, Burns LA, Dellinger EP, Garland J (2011). Guidelines for the prevention of intravascular catheter-related infections.. Clin Infect Dis.

[42] Flume PA, O’Sullivan BP, Robinson KA, Goss CH, Mogayzel PJJ (2007). Cystic fibrosis pulmonary guidelines: chronic medications for maintenance of lung health.. Am J Respir Crit Care Med.

[43] Lipsky BA, Berendt AR, Deery HG, Embil JM, Joseph WS (2004). Diagnosis and treatment of diabetic foot infections.. Clin Infect Dis.

[44] Cherkaoui A, Hibbs J, Emonet S, Tangomo M, Girard M (2010). Comparison of two matrix-assisted laser desorption ionization-time of flight mass spectrometry methods with conventional phenotypic identification for routine identification of bacteria to the species level.. J Clin Microbiol.

[45] Perkins DN, Pappin DJ, Creasy DM, Cottrell JS (1999). Probability-based protein identification by searching sequence databases using mass spectrometry data.. Electrophoresis.

[46] Dumitrescu O, Choudhury P, Boisset S, Badiou C, Bes M (2011). Beta-lactams interfering with PBP1 induce Panton-Valentine leukocidin expression by triggering *sar*A and *rot* global regulators of *Staphylococcus aureus*.. Antimicrob Agents Chemother.

[47] Benito Y, Lina G, Greenland T, Etienne J, Vandenesch F (1998). trans-complementation of a *Staphylococcus aureus agr* mutant by *Staphylococcus lugdunensis agr* RNAIII.. J Bacteriol.

[48] Walsh TR, Bolmstrom A, Qwarnstrom A, Ho P, Wootton M (2001). Evaluation of current methods for detection of staphylococci with reduced susceptibility to glycopeptides.. J Clin Microbiol.

[49] Hiramatsu K, Aritaka N, Hanaki H, Kawasaki S, Hosoda Y (1997). Dissemination in Japanese hospitals of strains of *Staphylococcus aureus* heterogeneously resistant to vancomycin.. Lancet.

[50] Wootton M, Howe RA, Hillman R, Walsh TR, Bennett PM (2001). A modified population analysis profile (PAP) method to detect hetero-resistance to vancomycin in *Staphylococcus aureus* in a UK hospital.. J Antimicrob Chemother.

[51] Monecke S, Slickers P, Ehricht R (2008). Assignment of *Staphylococcus aureus* isolates to clonal complexes based on microarray analysis and pattern recognition.. FEMS Immunol Med Microbiol.

