# Supplementary material for: Detection of Staphylococcus aureus Delta-Toxin Production by Whole-Cell MALDI-TOF Mass Spectrometry
Source: PLoS One. 2012 Jul 6;7(7):e40660. doi: 10.1371/journal.pone.0040660 (PMC3391297; doi:10.1371/journal.pone.0040660)
Supplement: Table S1 — Genotyping of 7 couples of Staphylococcus aureus isolates using microarrays. (PDF) [file pone.0040660.s002.pdf]

PATIENT NUMBER	DELTA STATUS	Strain reference	AGR Allele				Resistance or virulence genes (or alleles)																								
			agrI	agrII	agrIII	agrIV	mecA	tst	sea	seb	sec	sed	see	seg	seh	sei	sej	sek	sel	sem	sen	seo	sep	seq	ser	seu	lukSF-PV	lukM	etA	etB	etD
1	NEG	**ST20110473/BE10452343**	POS	NEG	NEG	NEG	NEG	NEG	NEG	NEG	NEG	NEG	NEG	NEG	NEG	NEG	NEG	NEG	NEG	NEG	NEG	NEG	NEG	NEG	NEG	NEG	NEG	NEG	NEG	NEG	NEG
	POS	**ST20110472/BE10404323**	POS	NEG	NEG	NEG	NEG	NEG	NEG	NEG	NEG	NEG	NEG	NEG	NEG	NEG	NEG	NEG	NEG	NEG	NEG	NEG	NEG	NEG	NEG	NEG	NEG	NEG	NEG	NEG	NEG
2	NEG	**ST20110474/BE10453198**	POS	NEG	NEG	NEG	NEG	NEG	NEG	NEG	NEG	NEG	NEG	NEG	NEG	NEG	NEG	NEG	NEG	NEG	NEG	NEG	POS	NEG	NEG	NEG	NEG	NEG	NEG	NEG	NEG
	POS	**ST20110475/BE10263218**	POS	NEG	NEG	NEG	NEG	NEG	NEG	NEG	NEG	NEG	NEG	NEG	NEG	NEG	NEG	NEG	NEG	NEG	NEG	NEG	POS	NEG	NEG	NEG	NEG	NEG	NEG	NEG	NEG
3	NEG	**ST20110479/BE10464190**	NEG	POS	NEG	NEG	NEG	NEG	NEG	NEG	NEG	NEG	NEG	NEG	NEG	NEG	NEG	NEG	NEG	NEG	NEG	NEG	NEG	NEG	NEG	NEG	NEG	NEG	POS	NEG	NEG
	POS	**ST20110481/BE09421183**	NEG	POS	NEG	NEG	NEG	NEG	NEG	NEG	NEG	NEG	NEG	NEG	NEG	NEG	NEG	NEG	NEG	NEG	NEG	NEG	NEG	NEG	NEG	NEG	NEG	NEG	POS	NEG	NEG
4	NEG	**ST20110488/BE10103306**	POS	NEG	NEG	NEG	POS	NEG	POS	NEG	NEG	NEG	NEG	NEG	NEG	NEG	NEG	NEG	NEG	NEG	NEG	NEG	NEG	NEG	NEG	NEG	NEG	NEG	NEG	NEG	NEG
	POS	**ST20110487/BE09503265**	POS	NEG	NEG	NEG	POS	NEG	POS	NEG	NEG	NEG	NEG	NEG	NEG	NEG	NEG	NEG	NEG	NEG	NEG	NEG	NEG	NEG	NEG	NEG	NEG	NEG	NEG	NEG	NEG
5	NEG	**ST20110489/BE11022376**	POS	NEG	NEG	NEG	POS	NEG	NEG	NEG	NEG	NEG	NEG	NEG	NEG	NEG	NEG	NEG	NEG	NEG	NEG	NEG	NEG	NEG	NEG	NEG	NEG	NEG	NEG	NEG	NEG
	POS	**ST20110490/BE10492196**	POS	NEG	NEG	NEG	POS	NEG	NEG	NEG	NEG	NEG	NEG	NEG	NEG	NEG	NEG	NEG	NEG	NEG	NEG	NEG	NEG	NEG	NEG	NEG	NEG	NEG	NEG	NEG	NEG
6	NEG	**ST20110493/BE10432239**	POS	NEG	NEG	NEG	POS	NEG	NEG	NEG	NEG	POS	NEG	NEG	NEG	NEG	POS	NEG	NEG	NEG	NEG	NEG	NEG	NEG	POS	NEG	NEG	NEG	NEG	NEG	NEG
	POS	**ST20110494/BE10323177**	POS	NEG	NEG	NEG	POS	NEG	NEG	NEG	NEG	POS	NEG	NEG	NEG	NEG	POS	NEG	NEG	NEG	NEG	NEG	NEG	NEG	POS	NEG	NEG	NEG	NEG	NEG	NEG
7	NEG	**ST20110491/BE11042334**	NEG	POS	NEG	NEG	NEG	NEG	NEG	NEG	NEG	NEG	NEG	POS	NEG	POS	NEG	NEG	NEG	POS	POS	POS	POS	NEG	NEG	POS	NEG	NEG	NEG	NEG	NEG
	POS	**ST20110651/BE10392351**	NEG	POS	NEG	NEG	NEG	NEG	NEG	NEG	NEG	NEG	NEG	POS	NEG	POS	NEG	NEG	NEG	POS	POS	POS	POS	NEG	NEG	POS	NEG	NEG	NEG	NEG	NEG

Strain reference	blaZ	blaI	blaR	ermA	ermB	ermC	linA	msrA	mefA	mpbBM	vatA	vatB	vga	vgaA	vgb	aacA-aphD	aadD	aphA3	sat	dfrA	far1	Q6GD50 (putat. fusidic acid resist.)
**ST20110473/BE10452343**	POS	POS	POS	NEG	NEG	NEG	NEG	NEG	NEG	NEG	NEG	NEG	NEG	NEG	NEG	NEG	NEG	NEG	NEG	NEG	NEG	NEG
**ST20110472/BE10404323**	POS	POS	POS	NEG	NEG	NEG	NEG	NEG	NEG	NEG	NEG	NEG	NEG	NEG	NEG	NEG	NEG	NEG	NEG	NEG	NEG	NEG
**ST20110474/BE10453198**	POS	POS	POS	NEG	NEG	NEG	NEG	NEG	NEG	NEG	NEG	NEG	NEG	NEG	NEG	NEG	NEG	NEG	NEG	NEG	NEG	NEG
**ST20110475/BE10263218**	POS	POS	POS	NEG	NEG	NEG	NEG	NEG	NEG	NEG	NEG	NEG	NEG	NEG	NEG	NEG	NEG	NEG	NEG	NEG	NEG	NEG
**ST20110479/BE10464190**	POS	POS	POS	NEG	NEG	NEG	NEG	NEG	NEG	AMB	NEG	NEG	NEG	NEG	NEG	NEG	NEG	NEG	NEG	NEG	NEG	NEG
**ST20110481/BE09421183**	POS	POS	POS	NEG	NEG	NEG	NEG	NEG	NEG	NEG	NEG	NEG	NEG	NEG	NEG	NEG	NEG	NEG	NEG	NEG	NEG	NEG
**ST20110488/BE10103306**	NEG	NEG	NEG	POS	NEG	NEG	NEG	NEG	NEG	NEG	NEG	NEG	NEG	NEG	NEG	NEG	POS	NEG	NEG	NEG	NEG	NEG
**ST20110487/BE09503265**	NEG	NEG	NEG	POS	NEG	NEG	NEG	NEG	NEG	NEG	NEG	NEG	NEG	NEG	NEG	NEG	POS	NEG	NEG	NEG	NEG	NEG
**ST20110489/BE11022376**	NEG	NEG	NEG	POS	NEG	NEG	NEG	NEG	NEG	NEG	NEG	NEG	NEG	NEG	NEG	AMB	NEG	AMB	POS	AMB	NEG	NEG
**ST20110490/BE10492196**	NEG	NEG	NEG	POS	NEG	NEG	NEG	NEG	NEG	NEG	NEG	NEG	NEG	NEG	NEG	POS	NEG	POS	POS	POS	NEG	NEG
**ST20110493/BE10432239**	POS	POS	POS	NEG	NEG	NEG	NEG	NEG	NEG	NEG	NEG	NEG	NEG	POS	NEG	NEG	POS	NEG	NEG	NEG	NEG	NEG
**ST20110494/BE10323177**	POS	POS	POS	NEG	NEG	NEG	NEG	NEG	NEG	NEG	NEG	NEG	NEG	POS	NEG	NEG	POS	NEG	NEG	NEG	NEG	NEG
**ST20110491/BE11042334**	POS	POS	POS	NEG	NEG	NEG	NEG	NEG	NEG	NEG	NEG	NEG	NEG	NEG	NEG	AMB	NEG	NEG	NEG	NEG	NEG	NEG
**ST20110651/BE10392351**	POS	POS	POS	NEG	NEG	NEG	NEG	NEG	NEG	NEG	NEG	NEG	NEG	NEG	NEG	NEG	NEG	NEG	NEG	NEG	NEG	NEG

Strain reference	mupR	tetK	tetM	cat	cfr	fexA	fosB	fosB-plasmid	qacA	qacC	tetEfflux	vanA	vanB	vanZ	MLST and agr group assignement
**ST20110473/BE10452343**	NEG	NEG	NEG	NEG	NEG	NEG	NEG	NEG	NEG	NEG	POS	NEG	NEG	NEG	agr_I/CC398 (score: 94.4 %)
**ST20110472/BE10404323**	NEG	NEG	NEG	NEG	NEG	NEG	NEG	NEG	NEG	NEG	POS	NEG	NEG	NEG	agr_I/CC398 (score: 92.6 %)
**ST20110474/BE10453198**	NEG	NEG	NEG	NEG	NEG	NEG	POS	NEG	NEG	NEG	POS	NEG	NEG	NEG	agr_I/CC8 (score: 92.2 %)
**ST20110475/BE10263218**	NEG	NEG	NEG	NEG	NEG	NEG	POS	NEG	NEG	NEG	POS	NEG	NEG	NEG	agr_I/CC8 (score: 92.4 %)
**ST20110479/BE10464190**	NEG	NEG	NEG	NEG	NEG	NEG	POS	AMB	NEG	NEG	POS	NEG	NEG	NEG	agr_II/CC15 (score: 91.0 %)
**ST20110481/BE09421183**	NEG	NEG	NEG	NEG	NEG	NEG	POS	NEG	NEG	NEG	POS	NEG	NEG	NEG	agr_II/CC15 (score: 93.8 %)
**ST20110488/BE10103306**	NEG	NEG	NEG	NEG	NEG	NEG	POS	NEG	NEG	NEG	POS	NEG	NEG	NEG	agr_I/CC8 (score: 90.0 %)
**ST20110487/BE09503265**	NEG	NEG	NEG	NEG	NEG	NEG	POS	AMB	NEG	NEG	POS	NEG	NEG	NEG	agr_I/CC8 (score: 89.0 %)
**ST20110489/BE11022376**	NEG	NEG	POS	NEG	NEG	NEG	POS	NEG	NEG	NEG	POS	NEG	NEG	NEG	agr_I/CC8 (score: 89.6 %)
**ST20110490/BE10492196**	NEG	NEG	POS	NEG	NEG	NEG	POS	NEG	NEG	NEG	POS	NEG	NEG	NEG	agr_I/CC8 (score: 91.5 %)
**ST20110493/BE10432239**	NEG	NEG	NEG	NEG	NEG	NEG	POS	AMB	NEG	NEG	POS	NEG	NEG	NEG	agr_I/CC8 (score: 93.6 %)
**ST20110494/BE10323177**	NEG	NEG	NEG	NEG	NEG	NEG	POS	AMB	NEG	NEG	POS	NEG	NEG	NEG	agr_I/CC8 (score: 92.8 %)
**ST20110491/BE11042334**	NEG	NEG	AMB	NEG	NEG	NEG	POS	AMB	NEG	NEG	POS	NEG	NEG	NEG	agr_II/CC5 (score: 91.8 %)
**ST20110651/BE10392351**	NEG	NEG	NEG	NEG	NEG	NEG	POS	NEG	NEG	NEG	POS	NEG	NEG	NEG	agr_II/CC5 (score: 92.4 %)

Table S1. Genotyping of 7 couples of *S.aureus* isolates using microarrays. Genotyping (Identibac, Alere Technologies) was performed by microarray probing of the delta-toxin positive isolates and the delta-toxin negative isolates previously cultured from the same patients in the preceding weeks. Probing results of representative genes out of the 185 genes and their allelic variants are shown as well as MLST assignement of each isolate.
