# Supplementary material for: Detection of Staphylococcus aureus Delta-Toxin Production by Whole-Cell MALDI-TOF Mass Spectrometry
Source: PLoS One. 2012 Jul 6;7(7):e40660. doi: 10.1371/journal.pone.0040660 (PMC3391297; doi:10.1371/journal.pone.0040660)
Supplement: Table S2 — Clinical characteristics of patients with chronic infections. (PDF) [file pone.0040660.s003.pdf]

Table S2. Clinical characteristics of patients with chronic infections.

Patient number	Clinical diagnosis	Delta toxin detection by WC-MALDI-TOF MS[a]
1	Chronic cutaneous abscess in diabetic hemodialysis patient	δ+[b]
2	Chronic bronchial suture infection in a lung-transplanted diabetic patient	δ+
3	Chronic lung infection in CF[c] patient	δ+
4	Chronic lung infection in CF patient	δ+
5	Chronic lung infection in CF patient	δ+
6	Chronic lung infection in CF patient	δ+
7	Chronic lung infection in CF patient	δ+
8	Chronic lung infection in CF patient	δ+
9	Chronic lung infection in CF patient	δ+
10	Chronic lung infection in CF patient	δ+
11	Chronic amputation-site infection in a diabetic patient	δ+
12	Diabetic foot infection	δ+
13	Chronic osteoarthritis of the left hand in a diabetic patient	δ+
14	Diabetic foot infection	δ+
15	Recurrent bacteremia in dialysis and diabetic patient	δ-[d]
16	Chronic lung infection in CF patient	δ-
17	Chronic lung infection in CF patient	δ-
18	Chronic lung infection in CF patient	δ-
19	Chronic lung infection in CF patient	δ-
20	Diabetic foot infection	δ-

[a]WC-MALDI-TOF MS: Whole-Cell Matrix Assisted Laser Desorption Ionization - Time-of-Flight mass spectrometry ; [b] δ+: detection of delta toxin ; [c]CF: cystic fibrosis ; [d]δ-: no detection of delta toxin.
